# Robot-assisted single-site compared with laparoscopic single-incision cholecystectomy for benign gallbladder disease: protocol for a randomized controlled trial

**DOI:** 10.1186/s12893-017-0206-1

**Published:** 2017-02-09

**Authors:** Lukasz Filip Grochola, Christopher Soll, Adrian Zehnder, Roland Wyss, Pascal Herzog, Stefan Breitenstein

**Affiliations:** Department of Surgery, Cantonal Hospital of Winterthur, Brauerstrasse 15, 8401 Winterthur, Switzerland

**Keywords:** Single Site, Single incision, Cholecystectomy, Robot-assisted laparoscopy, da Vinci, Surgeon’s Comfort, Stress load

## Abstract

**Background:**

Recent advances in robotic technology suggest that the utilization of the da Vinci Single-Site™ platform for cholecystectomy is safe, feasible and results in a shorter learning curve compared to conventional single-incision laparoscopic cholecystectomy. Moreover, the robot-assisted technology has been shown to reduce the surgeon’s stress load compared to standard single-incision laparoscopy in an experimental setup, suggesting an important advantage of the da Vinci platform. However, the above-mentioned observations are based solely on case series, case reports and experimental data, as high-quality clinical trials to demonstrate the benefits of the da Vinci Single-Site™ cholecystectomy have not been performed to date.

**Methods:**

This study addresses the question whether robot-assisted Single-Site™ cholecystectomy provides significant benefits over single-incision laparoscopic cholecystectomy in terms of surgeon’s stress load, while matching the standards of the conventional single-incision approach with regard to peri- and postoperative outcomes. It is designed as a single centre, single-blinded randomized controlled trial, which compares both surgical approaches with the primary endpoint surgeon’s physical and mental stress load at the time of surgery. In addition, the study aims to assess secondary endpoints such as operating time, conversion rates, additional trocar placement, intra-operative blood loss, length of hospital stay, costs of procedure, health-related quality of life, cosmesis and complications. Patients as well as ward staff are blinded until the 1^st^ postoperative year. Sample size calculation based on the results of a previously published experimental setup utilizing an estimated effect size of surgeon’s comfort of 0.8 (power of 0.8, alpha-error level of 0.05, error margin of 10–15%) resulted in a number of 30 randomized patients per arm.

**Discussion:**

The study is the first randomized controlled trial that compares the da Vinci Single Site™ platform to conventional laparoscopic approaches in cholecystectomy, one of the most frequently performed operations in general surgery.

**Trial registration:**

This trial is registered at clinicaltrials.gov (trial number: NCT02485392). Registered February 19, 2015.

## Background

Recent technological advances in both standard and robotic laparoscopic technology suggest that the surgical removal of the gallbladder can be done safely and effectively using the single port technique through one small periumbilical incision, which results in less scarring and possibly better cosmetic and clinical outcomes for the patient [1–6]. Despite beneficial patient-related results of laparoscopic single port cholecystectomy compared to the multiport laparoscopic operation, surgeons do not feel comfortable with the single port technology [6]. Hereby, the Da Vinci Single-Site™ technology provides several putative advantages over standard laparoscopic single-incision approaches such as the utilization of ergonomic, curved robot-assisted devices that preserve triangulation within the operative field, the reassignment of the crossing instruments to the opposite side, motion scaling and a stable 3D view of the operating field. These interesting features are thought to reduce eye strain, improve the ergonomic outcomes for surgeons and minimize both the mental and the physical workload associated with single-incision laparoscopy. Thus they possibly improve both surgical performance and work satisfaction, as well as eventually impact beneficially on patient well-being and outcome [3, 7].

Indeed, preliminary evidence from case reports and case–control studies suggests that the utilization of the da Vinci Single-Site™ technology for cholecystectomy is safe, feasible and results in a shorter learning curve compared to conventional single-incision laparoscopic cholecystectomy [1–4]. Hereby, some of the best evidence regarding robot-assisted single-site cholecystectomy comes from a study by Pietrabissa [3]. They conducted a multicenter, prospective, observational study, which included a total of over 100 robotic procedures. In this cohort, the authors reported no major intraoperative complications and only two of the cases were converted to an open procedure due to inflammation. All of the participating surgeons that had previous experience with single-incision laparoscopy cholecystectomy agreed that the robotic approach is easier than the single-incision laparoscopic technique.

Additional evidence for the benefits of the Da Vinci platform comes from a study by Schatte and colleagues [7]. Hereby, in a simulated experimental setup, the computer-aided technique significantly associated with cognitive and physical stress reduction. It has been therefore suggested that the utilization of the da Vinci robot system may provide several advantages to professionals who complain of fatigue and discomfort when conducting modern surgical procedures, such as single-incision laparoscopy. Indeed, surgeons are a group of health care professionals who are at risk of developing work-related musculoskeletal disorders because of many physical and psychosocial risk factors [8, 9]. Moreover, increases in technological complexity and equipment that is sometimes poorly adapted to new techniques have led surgeons to increasingly complain of fatigue when conducting modern surgical procedures [8, 10–13]. Importantly, it has been shown that a surgeon’s degree of burnout resulting from such fatigue and discomfort experienced at his or her workplace is strongly correlated with major medical errors, which in turn carry a great risk of affecting the patient’s well being and safety [14].

However, the described observations and putative advantages of the robotic technology are based solely on case series, case reports and experimental data, as high-quality clinical trials to demonstrate the benefits of the da Vinci Single-Site™ cholecystectomy have not been performed to date. The present study assesses in a randomized setting whether robot-assisted Single-Site™ cholecystectomy provides a significant benefit compared with single-incision laparoscopic cholecystectomy in terms of surgeon’s stress load as a primary endpoint.

## Methods/Design

### Study objectives

The study is designed as a single-blinded randomized clinical trial with an intention to treat analysis that compares Single-Site robot-assisted with single-incision laparoscopic cholecystectomy. It aims to to test the hypothesis that the robotic approach provides significant benefits in terms of a reduced surgeon’s stress load compared to the conventional single-incision approach, while matching its standards with regard to peri- and postoperative outcomes. The study directly compares both surgical approaches in patients with benign gallbladder disease.

### Endpoints and data collection

**Fig. 1:**
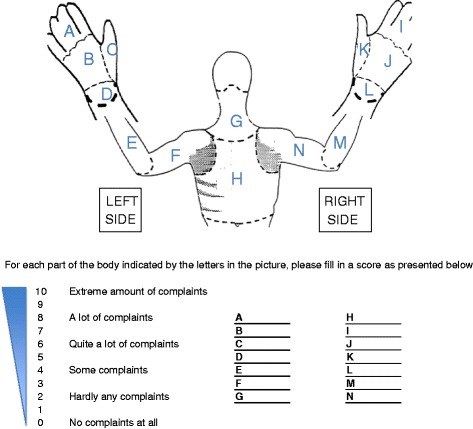
Local Experienced Discomfort (LED) Questionnaire visual analogue scale

**Fig. 2:**
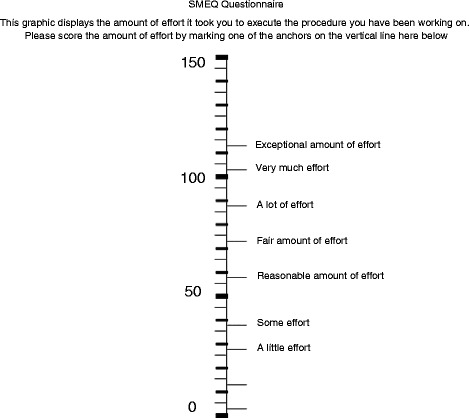
Subjective Mental Effort Questionnaire (SMEQ) visual analogue scale

**Fig. 3:**
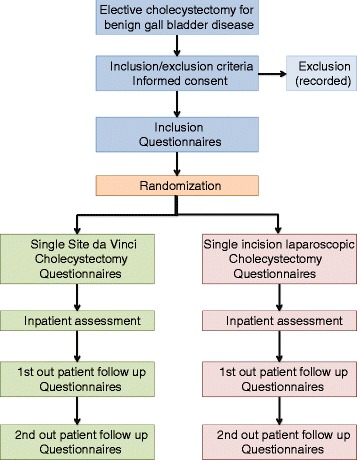
Study flowchart

The primary endpoint of the study is the surgeon’s physical and mental stress load at the time of surgery and is assessed by two validated visual analogue scales, specifically a modified Local Experienced Discomfort (LED, Fig. 1) and Subjective Mental Effort Questionnaire (SMEQ, Fig. 2) [7, 15]. Secondary endpoints include intra-operative blood loss, operating time, intra-operative conversion rate and additional trocar placement, complications, length of hospital stay, costs of procedure, Health-Related Quality of Life (HRQoL) and cosmesis. HRQoL and cosmesis will be assessed using the validated Gastrointestinal Quality of Life Index (GIQLI) and the Body Image Questionnaires (BIQ), respectively [16, 17]. The exact type and time of data collection is listed in detail in Table 1, the study flowchart is depicted in Fig. 3. All data will be recorded safely using the SecuTrial™ program. The estimated total duration of the study is 1.5 years, including the scheduled 1-month and 1-year postoperative follow-up visits. At the time of writing, the study is still active and the recruitment of patients has just been completed.

**Table 1:** Study outline

Schedule	Enrollment	Surgery	Inpatient assessment	1st Outpatient follow-up	2nd Outpatient follow-up^c^
Assessment No.	1	2	3	4	5
Time	–6 weeks to 0	0	up to 1 week^b^	1mo^b^	1 year
Informed consent	x				
Medical history	x				
In-/exclusion criteria	x				
Physical examination	x		x	x	(x) ^c^
Registration	x				
Imaging^a^	x				
Randomization	x				
Questionnaire 1: LED (surgeon)		x			
Questionnaire :2 SMEQ (surgeon)		x			
Duration of surgery		x			
Intraoperative blood loss		x			
Intraoperative conversion		x			
Evaluation of complications			x	x	x
Length of hospital stay			x		
Costs of procedure			x		
Questionnaire 3: HR-QoL (patient)	x			x	x
Questionnaire 4: Cosmesis (patient)				x	x

Each x signifies one test, report, or procedure

^a^USS and/or CT and/or MRI abdomen

^b^In case of AE, inpatient assessment and/or outpatient FU-visit might exceed the given time limit; All patients will be assessed until the day of discharge and 3–4 weeks after surgery

^c^The 2nd postoperative follow-up of the patient may either be done in the outpatient clinic or the assessement may be conducted by phone. In case the patient is seen in the outpatient clinic, the insurance company of the participant will not be charged by the hospital

### Inclusion criteria

Patients fulfilling the following inclusion criteria may be enrolled in the study:

- Patient compliance and geographic proximity allow proper preoperative check-up and postoperative follow-up
- Written informed consent given by the patient
- Women who are not breastfeeding and are not pregnant
- Age ≥18 years
- Symptomatic cholecystolithiasis
- Chronic cholecystitis
- Benign gallbladder polyps

### Exclusion criteria

The presence of one of the following criteria will lead to the exclusion of the subject:

- Significant systemic disease making the patient unsuitable for abdominal surgery (ASA IV and ASA V patients)
- Peritoneal carcinomatosis or other extensive metastatic disease
- Mental or organic disorders which could interfere with giving informed consent or receiving treatments
- Contraindications to pneumoperitoneum
- Suspicion of malignant disease
- Previous extensive upper abdominal surgery (upper gastrointestinal, hepato-pancreatico-biliary, colon, vascular and retroperitoneal surgery via laparotomy in the upper abdomen)
- Acute cholecystitis
- Emergency cholecystectomy
- Obesity II°-III° (BMI > 35.0 kg/m2)

All patients that have been excluded from participation will be recorded together with the reason for the exclusion.

### Randomization and blinding

Non-stratified block randomization (random block sizes two and four) will be used to achieve balance in the allocation of participants to both treatment arms and prevent a premature decoding of the randomization scheme. Hereby, the patient will not be informed about the group assignment until the last outpatient follow-up and only after he/she has completed and returned all required questionnaires (GIQLI and BIQ).

### Participating surgeons and clinic

The operation will be performed according to the group assignment by three senior surgeons only, who have extensive experience in both robotic Single-Site and conventional single-incision laparoscopy. All participating surgeons received a one-day training in both approaches (robotic Single Site and laparoscopic single incision cholecystectomy). All surgeries will be performed at the Department of Surgery at the Cantonal Hospital of Winterthur (Kantonsspital Winterthur) in Switzerland.

### Power of the study

Sample-size calculations are based on the results of the previously mentioned experimental setup by Schatte et al. [7], utilizing an estimated effect size of 0.8, at a power of 0.8 and an alpha-error level of 0.05, as well as considering a potential additional error margin of 10–15% of the calculations (G-Power 3.1 software, Heinrich-Heine University Duesseldorf/Germany). The sample-size calculations resulted in a number of 30 randomized patients per arm (60 patients in total).

### Statistical analysis

For descriptive statistics, median and mean with standard deviation will be used. For inductive statistics, the Chi-square and Fisher’s Test will be used to analyse contingency tables. In normally distributed data, a parametric two-sided Student’s T-Test (unpaired) to compare the data distributions in the two groups. In data that deviate from normal distribution, a two-tailed Mann–Whitney test will be applied to compare the data distributions in the two groups. All statistical analyses will be performed using SPSS (Version 22.0 and subsequent versions). P-values below 0.05 will be considered significant. No interim analyses are planned for the study.

### Ethics

The study will be carried out in accordance with principles enunciated in the current version of the Declaration of Helsinki, the guidelines of Good Clinical Practice (GCP) issued by ICH, and Swiss regulatory authority’s requirements. The principal investigator has completed a GCP course (modules 1–3). The study has been approved by the local ethics committee (Kantonale Ethikkommission Zürich, KEK-ZH-Nr. 2014–0114) and was registered at clinicaltrials.gov (trial number: NCT02485392).

### Surgical technique

The Single Site robot-assisted cholecystectomy using the da Vinci Si platform is performed according to the manufacturer´s instructions (Intuitive Surgical, Sunnyvale, CA, USA). The patient is placed in supine position under general anaesthesia, followed by surgical disinfection and sterile draping. A 2.0–2.5 cm midline intra-umbilical incision down to the level of the fascia is created using standard surgical technique. The fascia is incised and the abdomen is entered under direct vision. Subsequently, the fascial incision is enlarged to 2.5 cm and a finger sweep to check for adhesions is performed. Next, the surgeon inserts the Single-Site port using an atraumatic clamp. The Single-Site Port has a target anatomy arrow indicator and five lumens: four to hold cannulae (one 8.5 mm da Vinci Endoscope Port, two 5 mm Single-Site Instrument Arm Ports and one 5 mm Accessory Port) and one lumen for the insufflation adapter. After proper alignment of the Port, the table is placed in reverse Trendelenburg anatomy (~10–15°) under visual guidance (30° down endoscope) and docking is performed, whereby the patient cart approaches the patient from the right side in a ~45° angle. In contrast to the single incision conventional laparoscopic approach, the robotic technique utilizes ergonomic, curved robot-assisted laparoscopic devices that preserve triangulation within the operative field. The control of the crossing instruments is independently reassigned to the opposite side by the robotic software. The available accessories include curved scissors, hook, clip applier, needle driver, Cadiere grasper, Maryland retractor and also a suction-irrigation device. In addition, a laparoscopic grasper is inserted through the accessory cannula, which is utilized to retract the gallbladder fundus up and toward the patient’s right shoulder by the first assistant who is positioned on the left of the patient. After completion of the docking procedure, the console surgeon first dissects the Calot’s Triangle in a standard fashion, subsequently ligates and divides the cystic duct and artery, performs a gallbladder bed dissection and extracts the gallbladder specimen using a 10 mm EndoPouch Retriever™ instrument which is inserted through the accessory port after removal of the 5 mm assistant cannula. All instruments and the cannula are removed under visual guidance, the da Vinci Si platform is undocked and the Single Site port is removed together with the gallbladder and the specimen bag through the single incision. Lastly, the fascial defect is closed with 0 Vicryl and a subcuticular skin closure with 4/0 or 5/0 Monocryl is performed.

The single-incision laparoscopic cholecystectomy is performed as previously described with slight modifications [5]. In brief, a 2.0–2.5 cm midline intra-umbilical skin and fascia incision is performed and a SILS™ PT12 Port (Covidien Inc., Norwalk, California, USA) is introduced. This port obtains four openings: one for gas insufflation and three that can accommodate trocars ranging from sizes 5 – 12 mm. A 5-mm 45° long scope (HD EndoEye™, Olympus Europa Holding GmbH, Hamburg, Germany) is introduced through one of the openings in the SILS™ Port. The patient is then placed in an anti-Trendelenburg position. The fundus of the gallbladder is grasped and the EndoGrab™ (Virtual Ports Ltd., Caesarea, Israel) internally anchored retraction system is introduced (Endograsp roticulator™, Covidien Inc., Norwalk, California, USA). As an alternative, the TriPort Plus™ multitrocar port can be utilized (Olympus, Tokyo, Japan). The dissection of the Calot’s Triangle is done using a monopolar dissection hook. The cystic artery and duct are first dissected and then separately clipped with a standard 5 mm clip applicator (Covidien, Norwalk, California, USA). The gallbladder bed dissection is performed and once the gallbladder is free from the adjacent tissues, the 5 mm trocar is exchanged for a 10 mm trocar. An Endocatch bag (Endocatch Gold, 10 mm; Covidien Inc., Norwalk, California, USA) is inserted and the gallbladder is extracted. The umbilical fascia is closed using absorbable 0 Vicryl suture and a subcuticular skin closure with 4/0 or 5/0 Monocryl is performed.

## Discussion

The study is the first randomized controlled trial that compares the da Vinci Single Site™ platform to conventional laparoscopic approaches. The putative advantages of the robotic technique, such as a reduced surgeon’s physical and mental stressload, could lead to a further optimization and facilitation of laparoscopic approaches that might ultimately result in better clinical outcomes. In this study, the trial focuses on minimally-invasive cholecystectomy, one of the most frequently performed operations in general surgery. In fact, laparoscopic cholecystectomy remains an important focus of current surgical research efforts that aim to minimize the operative trauma and improve cosmesis without compromising safety and efficacy of this procedure. This trial compares two modern cholecystectomy techniques, which are likely to be adopted by the surgical community in the future on a larger scale, in a randomized controlled fashion. It utilizes a clear-cut study design with validated techniques to assess the study endpoints and an unbiased patient selection. The authors hope that it will help the current and future generation of surgeons to make an informed decision on which approach they will adopt to treat their patients with benign gallbladder disease.
